# Tolerability, safety and survival in patients with severe pulmonary arterial hypertension treated with intravenous epoprostenol (Veletri^®^): a prospective, 6-months, open label, observational, non-interventional study

**DOI:** 10.1186/s12931-022-02296-z

**Published:** 2023-01-18

**Authors:** Julia Degering, Benjamin Egenlauf, Satenik Harutyunova, Nicola Benjamin, Amina Salkić, Panagiota Xanthouli, Christina A. Eichstaedt, Rebekka Seeger, Olivier Sitbon, Ekkehard Grünig

**Affiliations:** 1 Centre for Pulmonary Hypertension, Thoraxklinik Heidelberg gGmbH at Heidelberg University Hospital, Röntgenstraße 1, 69126 Heidelberg, Germany; 2 Translational Lung Research Center Heidelberg (TLRC), Member of the German Center for Lung Research (DZL), Heidelberg, Germany; 3 Division of Rheumatology, Department of Medicine V, Hematology, Oncology and Rheumatology, University Hospital Heidelberg, Heidelberg, Germany; 4 Laboratory for Molecular Genetic Diagnostics, Institute of Human Genetics, Heidelberg University, Heidelberg, Germany; 5 Department of Respiratory Diseases, Bicêtre Hospital, Paris-Saclay University, Le Kremlin-Bicêtre, France

**Keywords:** Epoprostenol, Pulmonary hypertension, Veletri

## Abstract

**Background:**

Epoprostenol AS (Veletri^®^), a thermostable epoprostenol formulation, provides better drug stability and improved clinical use compared to previous epoprostenol formulations. This study aims to expand clinical experience in the use of Veletri^®^, especially regarding tolerability, safety and survival.

**Methods:**

Pulmonary arterial hypertension (PAH) patients at high risk despite pretreatment with at least double oral combination therapy and with clinical indication for epoprostenol (Veletri^®^) treatment were consecutively included in this prospective, open label, observational, non-interventional study. Clinical data were assessed at baseline, after 3 and 6 months. Adverse events (AEs) and serious adverse events (SAEs) were documented. Survival from initiation of Veletri^®^ was assessed at last patient out.

**Results:**

Fifteen patients (60 ± 13.7 years, WHO functional class III (n = 10) or IV (n = 5), severely impaired right ventricular function, mean pulmonary arterial pressure 54.8 ± 8.9 mmHg, mean pulmonary vascular resistance 4.4 ± 0.7 (median 3.8) Wood Units) were enrolled and treated with a mean dosage of 7.9 ± 3.9 (median 7.5) ng/kg/min. Eleven patients completed the study (treatment withdrawal n = 1, death n = 3). After a mean follow-up of 19.1 ± 13.5 (median 18.0) months, seven patients died and three were listed for lung transplantation. Seven AEs (nausea n = 3, diarrhea n = 1, flushing n = 2, headaches n = 1) and three SAEs (catheter infection n = 2, catheter occlusion n = 1) were related to Veletri^®^. The 1- and 2-year survival rates were 73.3% and 52.4%, respectively.

**Conclusions:**

The study showed that safety and tolerability of epoprostenol AS (Veletri^®^) was comparable to previous prostacyclin formulations and was feasible for most patients. The maximum tolerable dosage was lower than dosages reported in the literature. In future applications/trials the up-titration process should be pushing for higher dosages of epoprostenol in the occurrence of side effects, as the achievement of a high and effective dosage is crucial for the clinical benefit of the patients. Survival was as expected in these prevalent severely impaired patients.

*Trial registration* The study was registered in the EUPAS registry (EUPAS32492).

## Background

Chronic pulmonary hypertension (PH) is characterized by an increase of pulmonary vascular resistance and pulmonary arterial pressure, leading to right heart insufficiency and impaired prognosis [1, 2]. Dysregulation in prostacyclin synthesis with resulting deficiency of endogenous prostacyclin plays an important role in the pathogenesis of PH [3]. Prostacyclin has vasodilatory, anti-proliferative, anti-inflammatory and anti-thrombotic properties and is, therefore, an important target substance in PAH-specific therapy [4]. Epoprostenol was the first specific therapy approved for the treatment of PAH after showing positive effects on exercise capacity, key hemodynamic parameters, PAH symptoms and survival in otherwise treatment naïve patients [2, 5]. To date, epoprostenol is the only i.v. agent with high recommendation level for patients with severe PAH categorized in World Health Organization functional class (WHO-FC) III or IV [1, 6].

Epoprostenol, a synthetic prostacyclin, is chemically unstable at room temperature with a short biological half-life of 3–5 min. The agent can cause severe rebound PAH when infusion is interrupted abruptly [1]. Therefore, it requires continuous intravenous (i.v.) infusion via an in-dwelling central venous catheter and an external pump. Consequently, the application of epoprostenol is hampered by its handling, presenting a particular inconvenience for the patient to store and administer the medication [7].

Epoprostenol containing glycine and mannitol (GM) was originally approved for the use as a long-term continuous infusion in patients with PAH nearly 20 years ago in the USA; however, this formulation has limited stability at room temperature and requires the use of cooling or frequent medication changes during administration. With Veletri^®^, an improved bioequivalent formulation of intravenous epoprostenol has been developed containing the excipients arginine and sucrose (epoprostenol AS) (Veletri^®^, Caripul^®^). It provides better thermal stability with up to 72 h upon reconstitution depending on the concentration, is self-preserving and does not allow the growth of microorganisms [8]. Moreover, freshly prepared solutions with Veletri^®^ can be stored refrigerated for up to 8 days [7]. While former epoprostenol GM formulations such as Flolan^®^ required a specific diluent for reconstitution, Veletri^®^ can be reconstituted with either sterile water for injection or 0.9% sodium chloride injection [9].

Intravenous epoprostenol is used to treat adults with severe pulmonary arterial hypertension (PAH) (World Health Organization Group 1) and has been in use worldwide for decades. However, Veletri^®^ was under-used in clinical practice at the time the study started and still remains so to present day, despite available data suggesting a survival benefit if applied early as a part of combination therapy [10–12].

In Germany, Veletri^®^ is the only available epoprostenol-formulation with a safe 24 h drug-support. As intravenous treatment is still complex and experience in the administration, titration and handling of i.v. application systems is rare, this observational study aims to expand clinical experience and knowledge in the use of Veletri^®^ in patients with severe PAH, especially with regard to real-life data on tolerability, safety, clinical course of the disease and survival according to current clinical practice.

## Methods

### Study population and design

This was a 6-months, open label, observational, non-interventional prospective study to evaluate the use, safety and tolerability of intravenous epoprostenol AS (Veletri^®^). Fifteen patients with invasively diagnosed PAH by right heart catheterization (mean pulmonary arterial pressure ≥ 25 mmHg at rest, pulmonary arterial wedge pressure ≤ 15 mmHg) categorized in WHO functional class III and IV were consecutively included in this observational study between 03/2018 and 08/2020. All patients included received at least dual oral combination therapy with PDE-5 inhibitors and ERA and needed treatment escalation. Eligible patients for the study either had an unsatisfying long-term clinical response or were still in an intermediate or high-risk group despite dual combination treatment. Exclusion criteria comprised a known intolerance to epoprostenol or one of its excipients, pregnancy or lactation. Moreover, patients participating in any other clinical drug trial within 4 weeks prior to screening and/or patients scheduled to receive any investigational medicinal product during the course of this trial were not eligible. Patients provided written informed consent. The Ethics Committee of Heidelberg University had no objections against the conduct of this study (S-699/2017). The study complied with the Declaration of Helsinki in its current version. The study was registered in the EUPAS registry (EUPAS32492).

Patients were assessed according to routine clinical examinations at treatment initiation, after three and six months. Routine medical examinations comprised of medical history, physical examination, electrocardiogram (ECG), laboratory testing [including N-terminal pro brain natriuretic peptide (NT-proBNP)], 6-min walking test, echocardiography at rest, and right heart catheterization according to clinical practice. In order to assess quality of life, participants were asked to complete the SF-36 questionnaire. Survival and transplant-free survival was assessed at the 30 days follow-up visit after the patient’s last study examination visit.

A sample of 15 patients was aimed to be included into the study to gain insights into and collect data regarding the use, safety and tolerability of i.v. epoprostenol (Veletri^®^) treatment.

Data were collected for each patient at initiation of i.v. epoprostenol AS (Veletri^®^) and throughout the observational period from the existing medical records at any clinical visit on the respective case report form (CRF). Information was collected as available and per clinical practice visit schedule. Data were entered into a standardized CRF by study staff.

### Medication administration

To ensure a systematic approach, treating physicians were educated on recommendations for treatment initiation, dosage and titration, as well as common adverse events of intravenous epoprostenol.

Trial participants were hospitalized for treatment initiation in order to ensure continuous titration of Veletri^®^, adequate dose escalation and close monitoring. Differing to official dosage recommendations for rapid initiation of chronic infusion of i.v. epoprostenol (Veletri^®^), medication was started with 2 ng/kg/min (or lower if not tolerated) and increased in increments of 1–2 ng/kg/min every 24 h until a tolerance limit of the drug was established.

The dosage had to be decreased if dose-limiting pharmacologic effects occurred such as nausea, vomiting, hypotension, sepsis, headache, abdominal pain, or respiratory distress. During dose titration, the patients were observed by monitoring of standing and supine blood pressure and heart rate ensuring the tolerability of dosage increase.

In case of dose-limiting pharmacological events, a decrease in infusion rate was performed if deemed necessary; the dosage was decreased gradually in 1 ng/kg/min decrements every 15 min or longer until the dose-limiting effects resolved. Dosage was sustained if side effects were only mild and suspended spontaneously. Abrupt withdrawal of i.v. epoprostenol (Veletri^®^) or sudden large reductions in infusion rates were strictly avoided. Except for life-threatening situations (e.g., unconsciousness, collapse, etc.), infusion rates of i.v. epoprostenol (Veletri^®^) were only to be adjusted under the direction of a physician.

Veletri^®^, once prepared as directed, was administered by continuous i.v. infusion via a central venous catheter and an ambulatory infusion pump.

### Statistics

Data are presented as mean ± standard deviation and 95% confidence interval of the mean. Frequency tables are provided for qualitative data providing n and %. Safety and tolerability data of i.v. epoprostenol were collected and listed according to seriousness and relation to Veletri^®^ according to the judgement of the treating physician. The clinical course of the patients is presented descriptively. Kaplan–Meier survival analysis was performed. There was no imputation strategy for missing values.

## Results

### Study population (Table 1, Fig. 1)

**Table 1:** Baseline characteristics of the study cohort

	Study cohort (n = 15)	
	Mean ± standard deviation or n and %	
Sex (male/female)	3/12	
Age, years	59.9 ± 13.7	
Height, cm	169.5 ± 6.9	
Weight, kg	92.3 ± 31.3	
WHO Functional class		
III	10	(66.7%)
IV	5	(33.3%)
Diagnosis		
Idiopathic pulmonary arterial hypertension (PAH)	10	(66.6%)
Heritable PAH	1	(6.7%)
Connective tissue disease associated PAH	3	(20.0%)
Portopulmonary hypertension	1	(6.7%)
Time since initial diagnosis, months	45.4 ± 23.7	
Concomitant disease		
Cardiac		
Systemic arterial hypertension		(66.7%)
Coronary artery disease		(20.0%)
Atrial fibrillation		(13.3%)
Pulmonary		
History of pulmonary embolism		(26.7%)
Obstructive sleep apnea		(20.0%)
Chronic obstructive pulmonary disease		(6.7%)
Other		
Systemic sclerosis		(20.0%)
Hypothyreosis		(13.3%)
History of stroke		(13.3%)
Chronic renal insufficiency		(13.3%)
Hepatic cirrhosis		(6.7%)
History of splenectomy		(6.7%)
Diabetes mellitus		(6.7%)
PAH-targeted medication		
Endothelin receptor antagonists		
Bosentan	1	(6.7%)
Ambrisentan	4	(26.7%)
Macitentan	9	(60.0%)
Phosphodiesterase-5 inhibitors/ soluble guanylate cyclase stimulator		
Sildenafil	4	(26.7%)
Tadalafil	5	(33.3%)
Riociguat	6	(40.0%)
Prostanoids before starting Epoprostenol		
Treprostinil s.c	1	(6.7%)
Iloprost inhalative	2	(13.3%)
Iloprost i.v.	1	(6.7%)

**Fig. 1:**
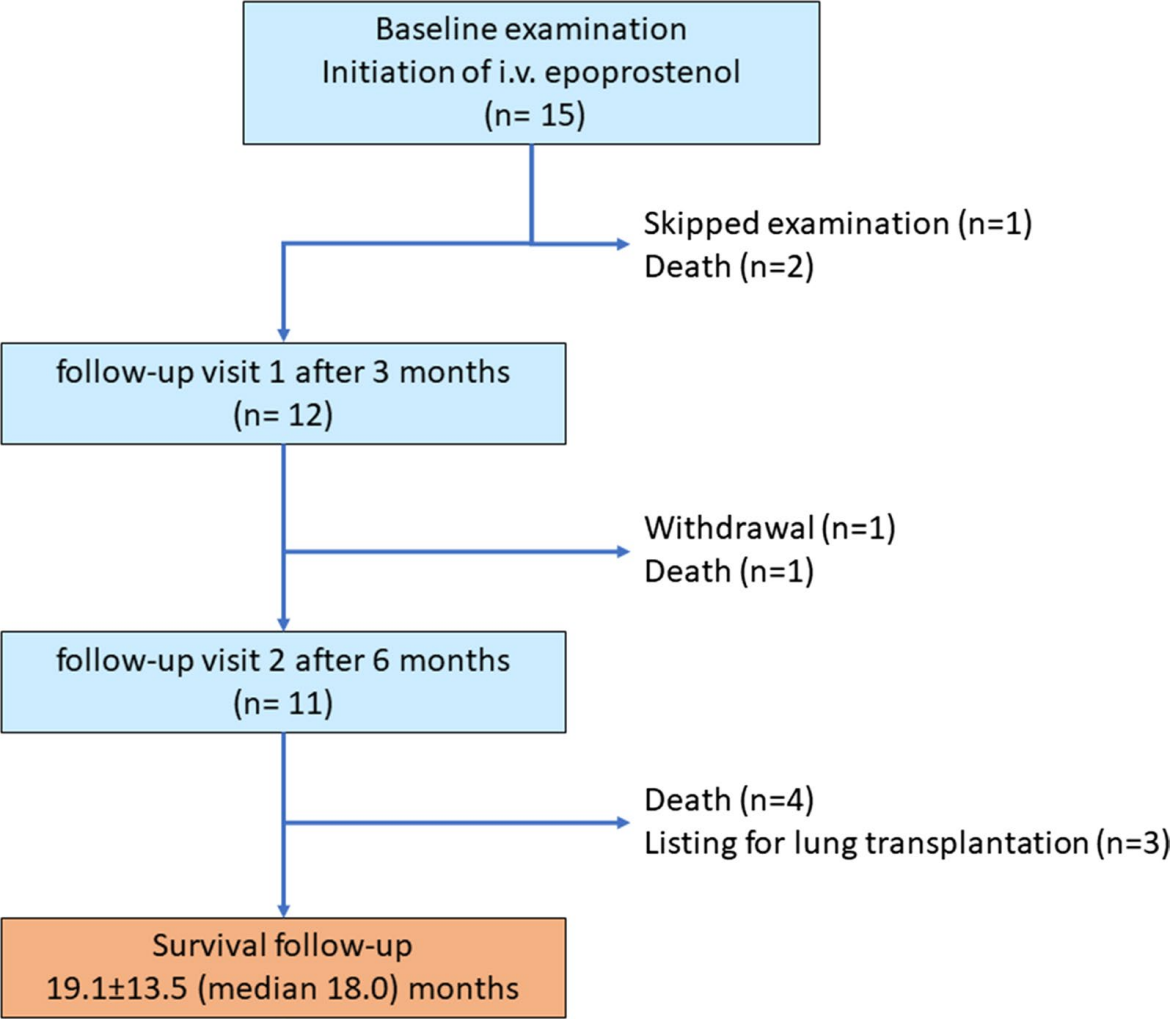
Study flow chart

The first patient was enrolled in March 2018. The last patient completed study treatment in March 2021. Overall, 15 patients (60 ± 13.7 years, 80% female, WHO functional class III–IV (Table 1), severely impaired right ventricular function, mean pulmonary arterial pressure 54.8 ± 8.9 mmHg, mean pulmonary vascular resistance 4.4 ± 0.7 (median 3.8) Wood Units) were enrolled. The majority had been diagnosed with idiopathic pulmonary arterial hypertension (IPAH) (66.6%) or PAH associated with connective tissue disease (20%, Table 1). The mean time from initial diagnosis to baseline was 45.4 ± 23.7 months. At study start, most patients were receiving dual oral combination treatment (n = 14) with PH-targeted medication in line with the eligibility criteria (Table 1). Four patients received additional prostacyclin analogues (inhalative iloprost n = 2, intravenous iloprost n = 1, subcutaneous treprostinil n = 1) before they were transitioned to Veletri^®^. Patients who had previously received other prostacyclin analogues such as treprostinil or iloprost were transitioned to epoprostenol AS (Veletri^®^). The reasons for transition to Veletri^®^ were insufficient effectiveness in the two patients with inhaled iloprost and problems with drug-supply in the patient with i.v. iloprost. The patient who formerly received treprostinil had repeated local infections nearby the application spot and therefore was switched to Veletri^®^. Prostanoid treatments were reduced over 3 days will uptitrating Veletri^®^.

According to the COMPERA approach, 14 patients were in the intermediate risk group and one in the high-risk group. Seven patients showed a REVEAL risk score ≥ 10.

Out of 15 included patients, seven patients died and three were listed for lung transplantation during the follow-up period. Three patients died within the study period of 6 months and four patients died during survival follow-up. Three patients were listed for lung transplantation during survival follow-up period.

Eleven patients completed the study (treatment withdrawal n = 1, death n = 3, Fig. 1).

During the study period of 6 months two patients died one month after treatment start, one 2½ months after treatment start. Deaths were due to multiple organ failure due to terminal cancer (n = 2) and due to right heart failure. Not all patients underwent all scheduled examinations at each study visit depending on their general condition. One patient withdrew treatment after three months due to medication intolerance and lack of treatment response with concomitant worsening of PH. This patient died approximately 3 months later. One patient skipped the follow-up visit at 3 months and participated at the 6-month visit.

### Use of Veletri®

**Fig. 2:**
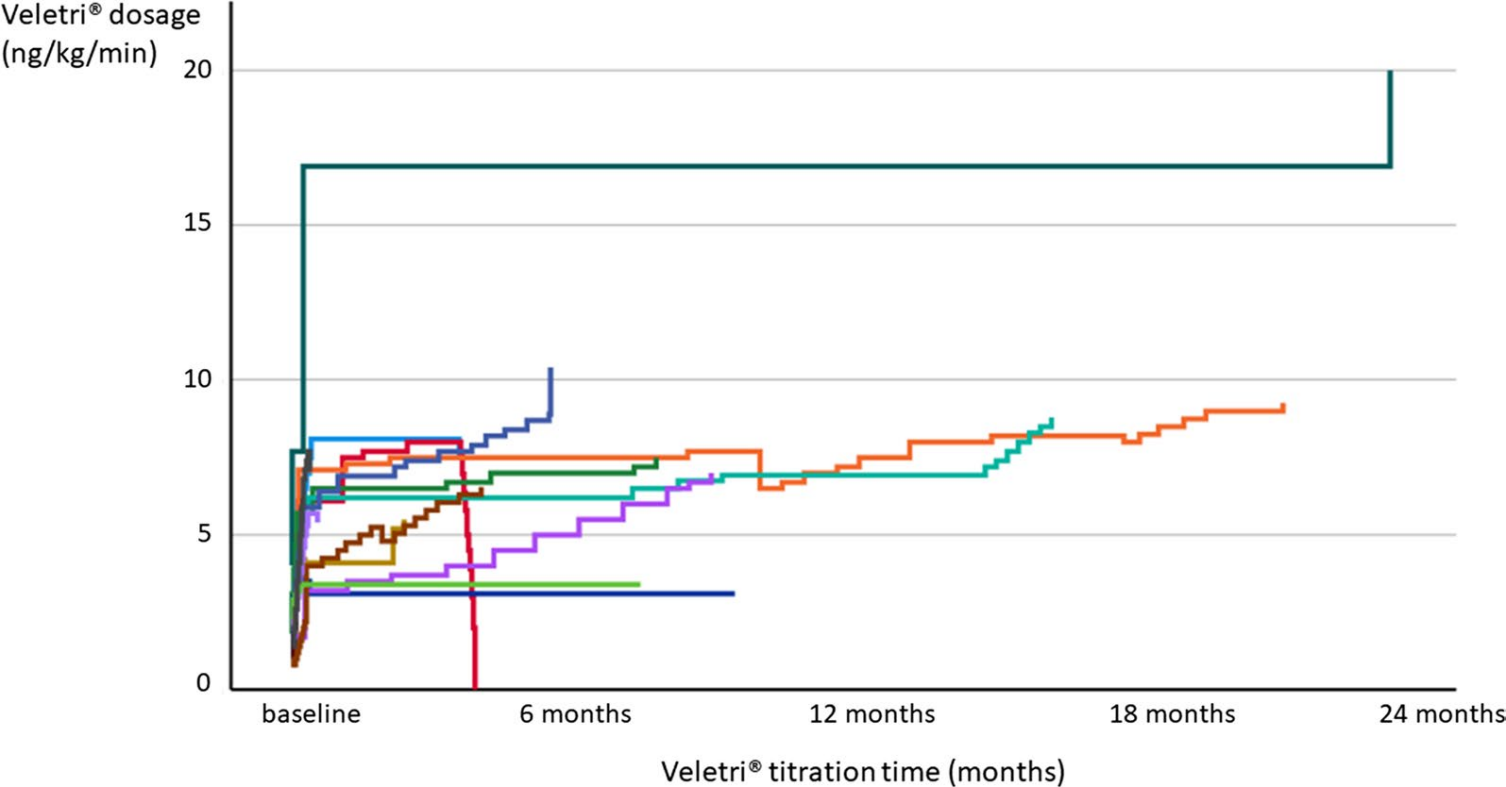
Dose titration of Veletri^®^

Veletri^®^ was titrated in-hospital for 7 ± 4 (1–12, median 7) days. Patients were treated with a mean maximum dosage of 7.9 ± 3.9 (3.4–20, median 7.5) ng/kg/min, most of them (12/15, 80%) with a dosage between 5 and 10 ng/kg/min. One patient, who received a markedly higher dosage of Veletri^®^ of 20 ng/kg/min, was formerly treated with Iloprost i.v. and could therefore be titrated faster and to a higher tolerable dosage (Fig. 2). Further dosages ranged from a maximum dosage of 3.4 ng/kg/min to a maximum dosage of 10.4 ng/kg/min.

Individual maintenance dosages of study treatment varied mainly between 5 and 10 ng/kg/min.

### Survival analysis

**Fig. 3:**
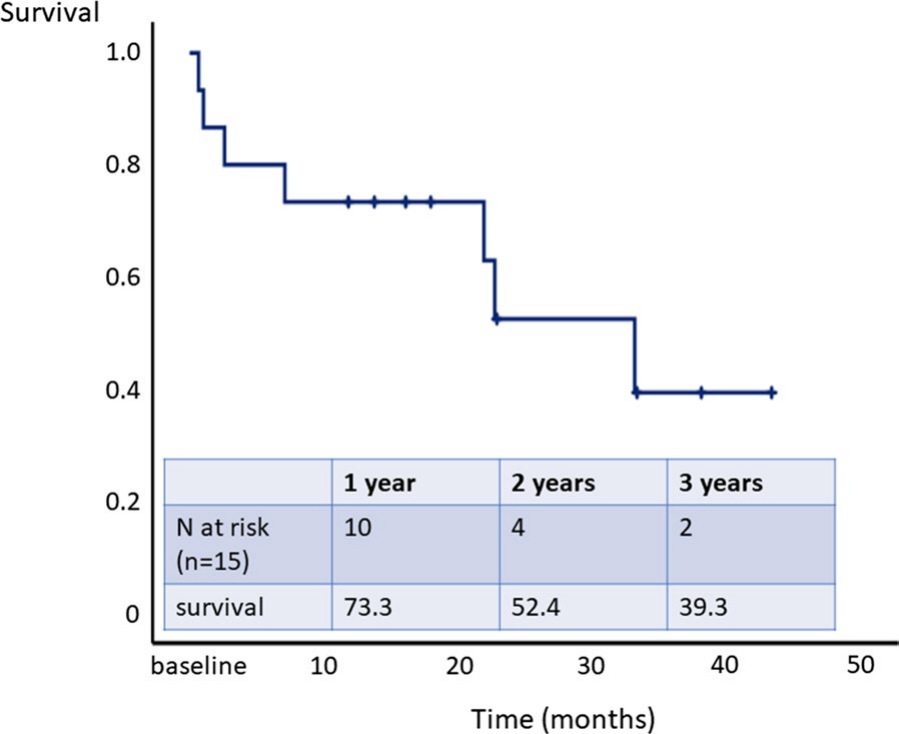
Survival analysis

After a mean follow-up of 19.1 ± 13.5 (median 18.0) months, seven patients died and three were listed for lung transplantation. The 1- and 2-year transplantation-free survival rates were 73.3% and 52.4%, respectively (Fig. 3). In addition to the three patients who died within the study period of 6 months, four patients died during follow-up. Reasons of death for patients dying during survival follow-up were acute renal failure (n = 2), cardiopulmonary failure (n = 1) and right heart failure (n = 1).

The 1- and 2-year transplantation-free survival rates were 73.3% and 52.4%.

### Safety of Veletri®

Sixteen serious adverse events (SAE) occurred in 12 patients. Three SAE were related to Veletri® treatment: catheter infection n = 2, catheter occlusion n = 1 (Table 2). The 10 SAEs unrelated to Veletri® comprised two cases of death 30 days and 77 days after treatment initiation, respectively, both considered unrelated to i.v. epoprostenol (Fig. 1). Another patient succumbed to PAH-associated right heart failure 19 days after i.v. epoprostenol initiation.

**Table 2:** Safety analysis

Adverse events	13	(93.3%)
Related to Veletri	6	(46.7%)
Nausea	3	(20.0%)
Flushing	2	(13.3%)
Diarrhea	1	(6.7%)
Headaches	1	(6.7%)
Not related to Veletri	7	(46.7%)
Feeling of thoracic pressure/pain	2	(13.3%)
Cardiac decompensation	1	(6.7%)
Hypoxia	1	(6.7%)
Itching	1	(6.7%)
Joint pain	1	(6.7%)
Edema	1	(6.7%)
Serious adverse events	16	(86.7%)
Related to Veletri	3	(20.0%)
Catheter infection	2	(13.3%)
Catheter occlusion	1	(6.7%)
Not related	13	(66.7%)
Cardiac decompensation	3	(20.0%)
Death	3	(20.0%)
Worsening of pulmonary hypertension	2	(13.3%)
Syncope	2	(13.3%)
Gynecologic bleeding	1	(6.7%)
Exanthema	1	(6.7%)
Erysipelas	1	(6.7%)

All three patients who died within 6 months from baseline had a history of cancer or suspected cancer (suspected pseudotumor cerebri and a mediastinal mass, mass in the right lower lobe of the lung, history of mamma carcinoma). Furthermore, all three patients had arterial hypertension, one patient showed coronary artery disease and one COPD II, GOLD B. All three patients presented with a NT-proBNP > 12.000 pg/l at baseline. Six-minute walking distance was 20 and 201 m; walking distance from one patient was not obtained. These patients were therefore classified as WHO functional class IV. Two out of the three patients died due to multiple organ failure, one due to right heart failure.

Out of the other four patients who died during long-term follow-up, two had concomitant systemic sclerosis, one patient a history of pulmonary embolism (though no CTEPH) and one patient suffered from liver cirrhosis. Two patients died due to acute renal failure, two due to suspected right heart failure.

Out of thirteen adverse events (AEs), 7 were related to Veletri^®^: nausea n = 3, flushing n = 2, diarrhea n = 1 and headaches n = 1 (Table 2) occurred within 120.1 ± 87.3 days of i.v. epoprostenol initiation. One of the patients had to discontinue epoprostenol treatment due to intolerance and therapy resistance after the 3-month follow-up visit. The other 6 epoprostenol-associated AEs were well manageable without hospitalization and could be improved or resolved without causing complications or treatment interruptions. Two patients experienced flushing as a dose-limiting AE, which resolved at a reduced dosage.

### Clinical course during Veletri^®^ treatment

Patients were assessed regarding their physical exercise capacity, hemodynamics, echocardiographic parameters, lung functional testing and quality of life. Mean changes of parameters are given in Table 3.

**Table 3:** Clinical course during the observation

Parameters	Baseline			Change to 3 months follow-up		Change to 6 months follow-up	
	Mean ± standard deviation		n	Mean ± standard deviation	n	Mean ± standard deviation	n
6-min walking distance, meters	267.7 ± 110.1		6	− 8.8 ± 62.4	6	4.7 ± 41.4	6
N-terminal pro brain natriuretic peptide, pg/l	4871.3 ± 6684.7		8	− 453.4 ± 5268.5	8	− 1159.6 ± 3446.2	8
SaO_2_ [%]	93.3 ± 2.4		12	− 0.5 ± 4.1	12	1.3 ± 3.1	11
Quality of Life (SF-36)							
Physical component score	34.4 ± 16.7		8	8.0 ± 17.0	8	6.6 ± 13.8	10
Mental component score	45.1 ± 25.1		8	2.5 ± 29.8	8	1.0 ± 15.5	10
Physical function	19.4 ± 15.5		8	5.0 ± 11.0	8	4.0 ± 13.3	10
Physical role performance	12.5 ± 35.4		8	25. ± 37.8	8	20 ± 36.9	10
Bodily pain	65.1 ± 18.9		8	9.3 ± 25.4	8	8.5 ± 16.1	10
General health	40.1 ± 17.4		8	− 2.3 ± 26.3	8	− 1.3 ± 17.6	10
Vitality	35.0 ± 24.9		8	3.8 ± 24.9	8	2.0 ± 16.0	10
Sovial functioning	51.6 ± 33.1		8	4.8 ± 37.0	8	− 1.3 ± 22.4	10
Emotional role performance	50.0 ± 53.5		8	0.0 ± 71.3	8	0.0 ± 47.1	10
Mental health	49.0 ± 20.3		8	6.5 ± 24.3	8	3.5 ± 19.9	10
Lung function							
FVC (L)	2.4 ± 0.6		7	0.2 ± 0.3	7	0.01 ± 0.4	7
FVC /FEV1[%]	73.3 ± 6.0		7	0.6 ± 4.2	7	2.7 ± 5.1	7
FEV1 [L]	1.9 ± 0.4		7	0.1 ± 0.1	7	0.04 ± 0.3	7
PEF [L/sec]	4.4 ± 1.5		7	0.2 ± 0.5	7	0.3 ± 0.6	7
DLCO [%]	50.1 ± 28.2		4	7.5 ± 8.3	4	0.13 ± 12.4	6
TLC [L]	5.2 ± 0.9		7	− 0.4 ± 0.9	7	− 0.2 ± 0.8	7
Residual volume [L]	2.7 ± 1.3		7	− 0.4 ± 0.9	7	0.1 ± 0.9	7
Echocardiography at rest							
sPAP [mmHg]	82.0 ± 15.7		10	− 9.2 ± 19.9	10	− 9.0 ± 25.8	10
RA area [cm^2^]	23.2 ± 5.8		11	0.8 ± 6.6	11	− 0.4 ± 6.3	10
RV area [cm^2^]	27.5 ± 6.2		11	− 1.5 ± 5.9	11	2.4 ± 5.8	10
TAPSE [cm]	1.98 ± 0.61		11	− 0.07 ± 0.35	11	0.09 ± 0.48	10
Tei-index	0.86 ± 0.32		8	− 0.15 ± 0.19	8	− 0.04 ± 0.23	7
Right heart catherization							
mPAP [mmHg]	58.3 ± 7.6		3			− 6.7 ± 11.5	3
sPAP [mmHg]	98.7 ± 11.8		3			− 11.7 ± 20.2	3
dPAP [mmHg]	36.6 ± 6.1		3			− 3.3 ± 5.8	3
PVR [dyn × s × cm^−5^]	8.2 ± 3.9		3			− 0.6 ± 1.1	3
mRAP [mmHg]	10.3 ± 4.6		3			− 4.0 ± 6.9	3
PAWP [mmHg]	10.3 ± 3.2		3			− 2.7 ± 4.6	3
Cardiac output [l/min]	6.4 ± 1.8		3			0.3 ± 0.5	3
Cardiac index [l × min × m^−2^]	2.8 ± 0.5		3			0.03 ± 0.23	3

DLCO: diffusing capacity of carbon monoxide, dPAP: diastolic pulmonary arterial pressure, FVC: forced vital capacity, FEV1: forced expiratory volume in the first second, mPAP: mean pulmonary arterial pressure, mRAP: mean right atrial pressure, NT-proBNP: n-terminal pro brain natriuretic peptide, PAWP: pulmonary arterial wedge pressure, PEF: peak expiratory flow, PVR: pulmonary vascular resistance, RA: right atrial, RV: right ventricular, SaO_2_: oxygen saturation, SF-36: short form health survey 36, sPAP: systolic pulmonary arterial pressure, TAPSE: tricuspid annular plane systolic excursion, TLC: total lung capacity, WHO: World Health Organization

Eight patients were able to perform a 6-min walking test at baseline. All patients had a 6-min walking distance (6MWD) below 440 m. Two had a walking distance below 165 m (ESC/ERS high-risk zone). Two patients showed a clinically relevant increase of 6MWD > 35 m, one patient had a decrease of 47 m (Table 3). The other patients remained stable. No patient showed a constant improvement of walking distance into the ESC/ERS low-risk category.

At baseline, five patients showed an increased NT-proBNP in the ESC/ERS intermediate risk zone of 300–1400 ng/ml, seven were in the high-risk group with NT-proBNP of > 1400 ng/ml. One patient improved from intermediate to low risk, one form high to intermediate risk. One patient showed worsening from intermediate to high risk category for NT-proBNP.

Out of 12 patients with at least two echocardiographic assessments, 9 showed a decrease of systolic pulmonary arterial pressure ranging between 5 and 50 mmHg.

In eight patients, right atrial area was in the intermediate risk group and in six patients in the high-risk group. Five patients showed an increase and five showed a decrease of right atrial area during the course of the study. One patient improved the ESC/ERS risk group for right atrial area from intermediate to low risk.

Right ventricular area decreased in eight and increased in four patients. Tricuspid annular plane systolic excursion increased in six (0.1–0.7 mm) and decreased in four patients (− 0.2 to − 0.8 mm).

Right ventricular function and WHO functional class remained stable in all patients during the course of the study.

Assessment of invasive hemodynamics by right heart catheterization could only be performed in three patients at baseline and follow-up. The other patients had a recently performed RHC before the beginning of the study. All three patients showed an increase in pulmonary vascular resistance.

A severely impaired quality of life [13] below 50 points at baseline was detected in eleven patients for the physical component scale and in nine patients for the mental component scale (Table 3).

Two patients had an increase of physical quality of life of 30 points in the SF-36 questionnaire. Mental health improved more than 10 points in 6 patients. Out of these patients, three showed a subsequent worsening of mental health. Two further patients decreased in mental health after baseline.

## Discussion

This study gives insight into safety, tolerability and clinical course during epoprostenol AS (Veletri^®^) treatment under real-world conditions. The treatment dosage of 7.9 ± 3.9 (3.4–20, median 7.5) ng/kg/min was comparable to dosages reported in the literature of median (range) dose at day 28 of 9.2 (8.0–15.0) ng/kg/min [14]. However, significantly higher dosages have been reported for long-term treatment with former epoprostenol formulations (for example Flolan®) of up to 30–40 ng/kg/min [15].

The highest dosage was reached in one patient who formerly received iloprost i.v. and switched to Veletri^®^, which is most likely due to a habituation effect of the drug requiring higher dosages in long-term use. The EPITOME-2 study suggests similar short-term safety and efficacy of Veletri^®^ in PAH patients stable on long-term Flolan^®^ therapy. However, considerably higher doses with a mean of 29.9 ± 15.1 ng/kg/min at baseline and 30.2 ± 15.0 ng/kg/min at three months were used [7]. In the controlled 12-week trial in PAH, the dose increased from a mean starting dose of 2.2 ng/kg/min. During the first 7 days of treatment, the dose was increased daily to a mean dose of 4.1 ng/kg/min on day 7 of treatment. At the end of week 12, the mean dose was 11.2 ng/kg/min. The mean incremental increase was 2–3 ng/kg/min every 3 weeks.

The safety profile in our study was comparable to data in the literature with nausea, diarrhea, flushing and headaches being among the most common adverse events related to the drug [2].

In terms of clinical efficacy, there was no general tendency of improvement in the parameters measured to assess exercise capacity (6MWD, Borg scale), quality of life (Short-form health survey 36; SF-36), hemodynamics, right heart size and function as well as lung function. WHO-FC also remained stable over the examination period.

Though no clinically relevant improvement was observed in this study, most patients remained stable under Veletri^®^ therapy. A stable clinical course under Veletri^®^ treatment has already been reported in the literature, this was however observed for patients switching from epoprostenol GM to epoprostenol AS [7, 15, 16]. Patients who changed from epoprostenol GM to epoprostenol AS have reported improved treatment convenience [17], while other quality of life parameters did not change. Though no change in treatment regimen was performed in this study, mental health increased in ten out of fifteen patients.

In patients with newly initiated epoprostenol, treatment effects include improvement of hemodynamics [5, 18, 19], improvement of physical exercise capacity [5, 20, 21] and improvement of quality of life [5, 22]. In our cohort, these effects could not be observed. This might be due to the severity of the disease at baseline, given that almost half of the study cohort died during the course of the study and follow-up. Possibly the given dose of Veletri^®^ was too low. However, all patients who died had underlying diseases which might have attributed to a higher mortality risk.

The majority of PH-related AEs (four out of six) were observed within 3 months after therapy initiation (mean: 120.2 ± 87.39 days, 95% CI: 28.6–211.8) and improved over the course, except for one death 19 days after Veletri^®^ initiation.

### Strengths and limitations of the study

This study was designed to generate insights into clinical practice, safety, tolerability and clinical course during treatment with Veletri^®^. An observational design is preferable when aiming to collect real-world data.

Results of this trial must be viewed considering its potential limitations due to the small number of participants and its non-interventional design, susceptible to loss to follow-up or withdrawals affecting results as well as significance. Treatment effects may not be accurately estimated due to missing values of patients who did not undergo all scheduled examinations at each visit. Moreover, one patient skipped the 3-month follow-up, another patient dropped out of the study after 3-month follow-up and three patients died during the 24-week study period due to causes likely unrelated to i.v. epoprostenol. Particularly changes in right heart catheterization endpoints could not be reliably determined as only three patients underwent this examination at the 6-month follow-up.

Perhaps, dose increments have been performed too low. During the in-hospital stay, epoprostenol had been started at 2 ng/kg/min and was increased by 1–2 ng/kg/min every 24 h until a dose of 10 ng/kg/min after 5 days was reached (in hospital). Afterwards, the dosage should have been increased by 1–2 ng/kg/min every 1–2 weeks to reach 16 ng/kg/min after 6–8 weeks. In most patients we did not reach this dosage. Possibly we did not increase the dosage enough after the in-hospital start of the therapy.

The study was aimed to expand clinical experience and knowledge. Dose titration is one important part which could be improved in future applications and which we learned from the PAH-centre in Hôpital Bicêtre in France.

Another limitation of this trial is the single-center nature of this trial. Thus, a selection bias cannot be excluded when recruiting patients. Moreover, this study was planned as single-arm, open-label study, which therefore lacks a control group for direct comparison.

Additionally, the use of questionnaires may have led to a bias, as, depending on their general condition, individuals may not be equally able to complete a questionnaire in turn affecting recorded scores.

Generally, the observation period of this trial was not long enough to reliably determine long-term treatment effects and survival in PAH patients. Further studies with a larger number of participants and an extended survival follow-up period are thus necessary to optimize titration, dosing and dose escalation and to determine efficacy in terms of (transplant-free) survival in patients with PAH in order to ultimately guide standardization of long-term therapy.

## Conclusions

The study shows that epoprostenol (Veletri^®^) treatment did not reveal new safety or tolerability aspects and was feasible in most patients. The survival rate was as expected in these severely impaired patients. In future applications/trials the up-titration process should be pushing for higher dosages of epoprostenol in the occurrence of side effects, as the achievement of a high and effective dosage is crucial for the clinical benefit of the patients.

## Data Availability

The dataset supporting the conclusions of this article is available upon reasonable request to the corresponding author.

## Abbreviations

6MWD: 6-Minute walking distance
AE: Adverse event
AS: Arginine and sucrose
CRF: Case report form
ECG: Electrocardiogram
GM: Glycine and mannitol
IPAH: Idiopathic pulmonary arterial hypertension PAH
i.v.: Intravenous
NT-proBNP: N-terminal pro brain natriuretic peptide
PAH: Pulmonary arterial hypertension
PH: Pulmonary hypertension
SAE: Serious adverse event
WHO-FC: World Health Organization functional class
